# HIV counselling and testing in secondary schools: What students want

**DOI:** 10.4102/sajhivmed.v16i1.390

**Published:** 2015-11-05

**Authors:** Estelle Lawrence, Patricia Struthers, Geert van Hove

**Affiliations:** 1 School of Public Health, University of the Western Cape, South Africa; 2 Department of Health, Provincial Government of the Western Cape, South Africa; 3 Department of Special Education, Ghent University, Belgium

## Abstract

**Background:**

HIV counselling and testing (HCT) is an essential element in the response to the HIV epidemic. There are still major research gaps about the best ways to provide HCT, especially to the youth, and school-based HCT is a model that has been suggested. To make HCT youth friendly and to enhance access to the service, the particular needs of the youth need to be addressed.

**Aim:**

To explore the expressed needs of students about school-based HCT service provision.

**Method:**

The study was conducted in 6 secondary schools in Cape Town where a mobile HCT service is provided by a non-governmental organisation. In each school, two mixed-gender focus groups were held, one with grades 8 and 9 students and one with grades 10 and 11. A total of 91 students aged 13–21 were involved. The focus groups were conducted in the students’ home language. All groups were audio-recorded, transcribed verbatim and translated into English.

**Results:**

Content data analysis was done and the following themes emerged: (1) Where the students want HCT to be done, (2) How they want HCT to be done and (3) Who should do the counselling. Most students want HCT to be provided in schools *on condition* that their fears and expressed needs are taken into account. They raised concerns regarding privacy and confidentiality, and expressed the need to be given information regarding HCT before testing is done. They wanted staff providing the service to be experienced and trained to work with youth, and they wanted students who tested positive to be followed up and supported.

**Conclusion:**

To increase youth utilisation of the HCT service, their expressed needs should be taken into account when developing a model for school-based HCT.

## Introduction

HIV counselling and testing (HCT) has been advocated as a critical entry point for care and treatment services, including prevention and clinical management of HIV-related illnesses, and psychosocial support.^1,2,3,4,5^ However, despite a national HCT campaign from 2010 to 2012 in South Africa, only 50.6% of youth aged 15–24 years reported testing, compared with 78.2% of adults aged 25–49 years.^6^

As part of the national HCT campaign, the South African Departments of Basic Education and Health announced that they intended to launch an HCT campaign specifically targeting secondary school students.^7^ This announcement was met with widespread concern from child rights, human rights and AIDS organisations, who argued that the school setting was not conducive to providing HCT in a way that does not violate the rights of youth. Owing to these legal and ethical concerns, the Department of Basic Education put the campaign on hold until policies guiding HCT in schools could be developed.

Little research has been done on the school-based model of providing HCT, and it is not known if this model meets the needs of youth. School-based models have been described in Uganda, where the Kitovu Mission Hospital has provided a mobile HCT service in schools,^8^ and the Tholulwazi Uzivikele HCT programme in Manguzi, South Africa, where HCT was offered in schools; drama used to raise awareness and encourage testing amongst the students.^9^ However, these studies were descriptive and did not evaluate the acceptability or effectiveness of the programmes. The objective of the present study was to explore the expressed needs of secondary school students regarding HCT provision at schools, and to add the voice of youth to the discussion regarding school-based HCT.

## Methodology

### Setting

The study was done with a non-governmental organisation (NGO) in Cape Town, South Africa, which had been providing a school-based HCT service since 2005 (before the South African government announced the HCT campaign) in an attempt to make HCT more accessible to youth. The NGO provided mobile school-based HCT to schools that requested their service. With consent, students were tested class by class in the school hall (or similar space). They received individual pre- and post-test counselling and were tested by a nurse using a finger-prick rapid HIV test. Results were given 15 minutes after the test, and students who were found to be HIV-positive were referred to a health facility of their choice for further management. The team usually tested for a number of days at one school, depending on the size of the school and the demand for testing (personal communication with NGO project manager, 2010).

### Sample

Six public secondary schools were purposively selected from a list of schools where the NGO provided the school-based HCT service. The six schools were selected so as to obtain the views of youth from diverse backgrounds in terms of their home language, ethnicity and school quintile.

One school was selected from quintiles 1–4 (schools A, B, C and D) and two schools from quintile 5 (schools E and F) because, even though they were in the same quintile, the socio-economic status and racial backgrounds of the students at the two quintile 5 schools were very different (Table 1). At the time of sampling, the NGO was not doing HCT at any quintile 1 secondary schools; however, one quintile 1 school was selected (school A), to gather the perspective of students who had not previously been exposed to HCT at school.

**TABLE 1:** Profile of selected schools.

School	Quintile†	HCT taken place at school	Home language	Racial groups‡
A	1	No	Xhosa	Black
B	2	Yes	Xhosa	Black
C	3	Yes	Xhosa	Black
D	4	Yes	English/Afrikaans	Mixed race
E	5	Yes	English	Black, mixed race
F	5	Yes	English	White, black, mixed race, Indian

HCT, HIV counselling and testing.

†, The South African Department of Education classifies schools according to relative poverty in five categories called quintiles. The quintile score is based on the national census data of the school catchment area and depends on income, unemployment rate and level of education. Schools from the poorest catchment areas are in quintile one, and from the least poor in quintile five.

‡, During the apartheid era, the South African government classified people into four major racial groups (black/African, mixed race, Indian/Asian and white/European). Post apartheid, many South Africans still identify themselves and others according to these groups.

The Life Orientation (‘the study of the self in relation to others and to society. It addresses skills, knowledge, and values about the self, the environment, responsible citizenship, a healthy and productive life, social engagement, recreation and physical activity, careers and career choices’^10^) teacher of each school was informed about the study purpose and asked to select students for two focus-group discussions (FGDs) at each school, one with grades 8 and 9 students and one with grades 10 and 11 students. The inclusion criteria were: students who were confident in groups; students who were able to express themselves well; and students who were not all leaders. The Life Orientation teacher was asked to select students from diverse population groups (where possible), and to ensure a similar number of male and female participants.

### Data collection

In each of the six schools, two FGDs were conducted with students, with a total of 91 student participants (Table 2). All the groups were mixed-gender except for the grades 10 and 11 group at school D. None of the male students arrived for the FGD, even though they had signed consent to participate. Their reason for not attending was not established; the Life Orientation teacher felt that it was because the FGD was held after school hours.

**TABLE 2:** Sex and age of focus-group discussions participants.

School	Grades 8 and 9							Grades 10 and 11						
	Male							Female							Total		Age (years)	Male							Female							Total							Age (years)
	n	%	n	%		Range	Mean	n	%	n	%		Range	Mean
A	2	29	5	71	7	14–16	15	4	44	5	56	9	16–19	17
B	3	29	4	71	7	14–16	15	4	50	4	50	8	16–20	18
C	4	44	5	56	9	13–16	15	3	50	3	50	6	17–21	18
D	7	70	3	30	10	14–15	14	0	0	5	100	5	17–18	17
E	3	33	5	63	8	13–15	14	1	17	5	83	6	15–17	16
F	4	50	4	50	8	13–15	14	4	50	4	50	8	16–17	16
**Total**	**23**	**47**	**26**	**53**	**49**	**13–16**	**14.5**	**16**	**38**	**26**	**62**	**42**	**15–21**	**17**

FGD, Focus-group discussion.

Each FGD was conducted in the students’ home language by a trained facilitator, who focused on the discussion process (guiding the discussion and encouraging equal participation by all students), and an assistant who took comprehensive notes and documented non-verbal communication. The FGDs were held in one of the classrooms at the school involved. At the beginning of the FGD, a role-play was used to trigger the students’ thinking. Students were divided into two teams and were asked to create a role-play which illustrated what they think happens when students go for school-based HCT. Once the teams had watched each other's role-play, the two teams came together for the FGD. A set of questions linked to the role-plays guided the discussion:

- What do you think will make students want to use this testing service?
- What do you think will make students *not* want to use this testing service?
- What would you like to experience when you go for HCT at school?
- Do you think the school is a good place to do HCT? Why do you say that?

Ethical clearance was obtained from the University of the Western Cape Senate Ethics Committee, and permission to conduct the study was given by the Western Cape Education Department as well as the schools’ principals. Students ≥ 18 years old gave written informed consent to participate; those < 18 years gave assent, and written informed consent was obtained from their parents or guardians.

### Data analysis strategy

Transcripts of the audio-recordings of the FGDs were loaded into Nvivo 8 and an inductive approach of content analysis was used to analyse the data. This involved close reading of the text, identifying segments related to the research question, coding these texts and assigning them to categories. A process of refining and revising the categories continued until three main themes emerged: (1) Where we (the students) want HCT to be done, (2) How we want HCT to be done and (3) Who should do the counselling (Table 3).

**TABLE 3:** Themes which emerged during data analysis.

Subcategory	Category	Theme
School accessible	At school	Where we want HCT to be done
School convenient		
Will not be seen by community members		
Will not be seen by other students	At clinic	
Service at clinic of better quality		
More private	At home	
Concern that others will assume HIV+	We do not want to be seen going for HCT	How we want HCT to be done
Concern that others will assume sexually active		
Do not want to be seen if upset by positive result	We want HCT to be done in a place that provides privacy	
Do not want others to hear result being given		
Need to know benefits of HCT	We want information about HCT before testing takes place	
Need to know procedure of HCT		
Do not want paper trail	We want confidentiality guaranteed	
Want counsellors to promise confidentiality		
Discomfort with being asked questions about sexual activity	We do not want to be asked too many questions	
Emotional support if positive	We want those who test positive to be supported	
Support with follow-up treatment		
Friendly service providers	We want service providers whom we can easily communicate with	Who should do the HCT
Non-judgemental service providers		
Patient service providers		
Experienced service providers	We want service providers who are competent to work with youth	
Specially trained service providers		
Service providers whom youth can relate to	We want service providers who are ‘young’	
Service providers who can give advice and support		

HCT, HIV counselling and testing.

To improve the accuracy and validity of the coding and interpretation of the data, an independent researcher was asked to code the data, and member checks were done with the students who participated in the FGDs.

## Results

The students’ expressed needs were similar across schools, gender, age and grade, except where mentioned in the findings. There was also no difference between responses from the school where no HCT had ever taken place, and those schools where it had taken place. The responses from the all-female group were similar to the mixed-gender groups.

### Theme 1: Where we want HIV counselling and testing to be done

When asked whether they thought their school was a good place to have testing done, most students stated that they would prefer to have HCT provided at their school during school hours. They explained that they would not find time after school to attend a clinic for testing. They also found it difficult to get to clinics because they were often located far from where they live:

‘It is easy if it's done here at school, ’cause I would have never gone for testing if it wasn’t.’ (female student, age 14)

Students also would prefer testing at school rather than at a clinic, as they feared being seen at the clinic by community members who might tell their parents that they had gone for testing:

‘… [*T*]he other woman is going to say, “Oh no! Your child was at the clinic! What was she doing there? She was sitting on the other side for people who go to test.”’ (female student, age 17)

A few students felt that they would prefer being tested at a clinic. Reasons included not wanting to be seen by other students when going for testing; concerns about confidentiality at school; and perceiving the quality of service at the clinic as being better. One student felt that he would prefer being tested at home, as it was more private.

### Theme 2: How we want HIV counselling and testing to be done

When stating that they would like to be tested at their school, students invariably added the proviso that it must be done ‘in a right way’.

#### We do not want to be seen going for HIV counselling and testing

When describing what they meant by ‘in a right way’, students said that they did not want other students and teachers to know that they were going for HCT. They had huge concerns about what their peers and teachers would think of them if they were to be seen going for testing:

‘Young people, they don’t want this kinda thing [*going for HCT*] to be seen by others.’ (female student, age 17)

‘As a young person … something that is extremely important for us, is what people think about us.’ (female student, age 16)

Reasons for concern about ‘What people think’ differed amongst the schools, which might have been linked to the students’ racial groupings. Students from schools D (only mixed race students), E and F (students from various racial groups) were concerned that others would assume that they were sexually active if they went for testing. In contrast, students from schools A, B and C, where all the students were black, were concerned that, if they went for testing, others would assume that they were HIV-positive:

‘…[*b*]lack people … if you do go for a test, it's not because you want to know your status. It's because you are definitely [HIV] positive’. (female student, age 15)

A 15-year-old black male student at school A pointed out that male students do not want female students to see them go for testing, because it would be assumed that they were HIV-positive, and suggested that male students be tested on a separate day from female students:

‘If you queuing there and you see a girl that you like … you are going to get shy if you are also there to test. You are going to think, “No, no.”’

A 16-year-old black female student in the same FGD corroborated what he said:

‘If maybe [*he*] goes in there to test, most girls are going to distance themselves from him, thinking that he is already positive … that is how most girls think.’

To avoid being seen, students suggested going for testing one at a time rather than class by class in the school hall (some suggested an appointment system).

#### We want HIV counselling and testing to be done in a place that provides privacy

The need for a space that provided visual and auditory privacy during the HCT process was consistently mentioned. Students specifically did not want to be tested in the school hall (the site where HCT took place at each of the schools). They explained that tents or cubicles were erected in the hall to try to provide visual and auditory privacy. However, they felt that the degree of privacy provided was not adequate, and preferred counselling in separate rooms:

‘About the privacy, I do think they need a room, like different rooms for each counsellor … this cubicle thing is just too open. It's just too public … The whole grade [is] behind you, like a few meters behind you … and there you’ve just heard that you’re positive, and the person [*is*] right behind you.’ (male student, age 16)

After receiving their results, they had to face students waiting to be tested, and were afraid that they would not be able to hide the fact if they had just been told that they were HIV-positive:

‘When I get told that I am positive, even if I keep quiet about it, I will have a facial expression that I make. If I am coming out, they are going to first look at me in the face, what facial expression I’m going to make, and then they know that if I am crying I am HIV-positive, and if I am smiling they know that I am not.’ (female student, age 16)

Some students proposed that the counselling area should have a separate exit so that, after receiving their results, students did not have to face other students waiting in the queue.

#### We want information about HIV counselling and testing before testing takes place

Students attributed an ‘it won’t happen to me’ attitude towards HIV as a reason for not testing. They believed that many students thought HIV and AIDS only affected older people, and thought it was necessary for students to be informed about the benefits, importance and procedure of HCT prior to testing taking place. Others felt that the fear of testing positive was a barrier to testing, and suggested that information be given about what to do if one should test HIV-positive, as they felt that this would reassure students and alleviate some of these fears.

#### We want confidentiality guaranteed

Students mentioned confidentiality as an important part of doing the testing ‘in the right way’. They wanted staff to promise confidentiality up front, and preferred that only counsellors (not nursing and administrative staff) knew the HIV results and that no ‘paper trail’ was kept of results.

#### We do not want to be asked ‘too many questions’

In many of the FGDs, students said that they felt uncomfortable with being asked questions about sexual activity. They considered these questions to be ’private’ and wanted their privacy respected:

‘Sometimes they ask too much questions. Maybe they ask you now, “Are you sexually active?” Now you say, “Yes,” then they ask you, “When last did you have sex?” You had sex yesterday last … you dunno how to tell her, because she's a big person [*an adult*] and you telling her now.’ (female student, age 17)

#### We want those who test positive to be supported

Students felt it important that those who test positive be followed up and given support by the NGO. They wanted assistance with disclosing to family members, emotional support and encouragement with adherence if needing to take antiretrovirals:

‘If they say you are positive … they should do check-ups to see … if you are coping.’ (female student, age 17)

### Theme 3: Who should do the counselling

#### We want to be counselled by staff whom we feel we can easily communicate with

Students said they wanted the counsellors to be people who are easy to talk to and who would make them feel comfortable. They felt it was essential that the counsellors were friendly, non-judgemental and treated youth with respect. They wanted the counsellors to be patient, and expressed the need to have enough time with the counsellor to have things explained properly, and to have the opportunity to ask questions.

#### We want to be counselled by staff who are competent to work with youth

It was important to students that the counsellor was experienced, behaved professionally and had received training to provide a youth-friendly health service (YFHS);

‘The counsellor should be taught … about teenagers of today and how they function.’ (female student, age 17)

#### We want HIV counselling and testing to be done by someone who is ‘young’

Some students preferred the counsellor to be young (they defined ‘young’ as someone between the ages of 20 and 30). They felt that a younger counsellor would be easier to relate to, and would better understand them (‘Older people don’t know what we are going through.’ [female student, age 17]). Some students preferred an older counsellor (they defined ‘older’ as someone in their 30s). Those who preferred an older counsellor felt that an older person would have more knowledge and experience, and therefore be better able to give advice. Students in one FGD favoured a counsellor under the age of 20 years, whereas other students in the same FGD specified that, though they preferred a younger counsellor, they did not want to be counselled by someone their own age, as they felt that their peers would only have as much knowledge as they did.

## Discussion and implications

Findings suggest that most students (across socio-economic groups, racial groups, gender, age and grade) consider school-based HCT to be more accessible and convenient than a health facility-based HCT service, which is similar to the findings of Henry-Reid et al.,^11^ who proposed that school-based HCT services are more accessible and acceptable to youth than other formal health settings; and Madiba and Mokgatle^12^ who found that the acceptability of HCT at schools in Gauteng and North-West Provinces, South Africa, was high.

The students made it clear, however, that if HCT were to be offered at school, it has to be provided in a manner that takes into account their needs. They had very specific ideas about what they wanted and did not want. Most of their expressed needs were based on fear: fear of being seen going for testing, fear of testing positive, fear of their HIV-positive status being known and the stigma associated with it, fear of not being supported if they tested positive, and fear of being judged and not being understood. In fact, the few students who preferred testing at a health facility or at home favoured these settings owing to concerns regarding privacy and confidentiality at school.

Similar fears about being seen going for HCT have been expressed by African youth in other studies.^12,13,14,15^ The need for auditory and visual privacy during counselling was also expressed by adolescents attending clinics that were part of a YFHS project in Zambia.^16^ The guarantee of confidentiality was also mentioned as an important need by youth in studies done in other African countries investigating youth needs regarding YFHS.^16,17^ The students’ concerns about not receiving ongoing counselling for dealing with a positive diagnosis and to assist with access to treatment is echoed in the findings of MacPhail et al.^15^ in their study with adolescents in two South African townships. Students’ anxieties that they would be judged, disrespected and not understood by HCT service providers have also been described in previous studies.^15,17,18,19^ Similarly, in a systematic review, healthcare providers’ attitudes, communication skills and competency were cited by youth as indicators of youth-friendly health care.^20^

The expressed needs of students regarding school-based HCT, as described in the present study, coincide with some of the characteristics of a YFHS described by the World Health Organization (WHO)^21^; namely, that the service should have policies that guarantee privacy and confidentiality; that service providers are easy to relate to, non-judgemental, have good interpersonal and communication skills, and are competent to work with youth; and that testing facilities offer privacy. To be noted is the fact that students in the present study did not mention youth involvement in service provision and assessment. Similarly, in the survey carried out by Erulkar et al.^18^ amongst Kenyan and Zimbabwean youth, youth involvement was also not mentioned as a priority for providing a YFHS.

A number of limitations exist in the present study. All the groups, except one, were of mixed-gender, and all the facilitators were female, which might have affected responses by students. However, one group comprised only female students and their responses were similar to the responses in the mixed-gender groups. Also, all the schools were urban schools; therefore, findings might be different with students from rural schools. Because of these limitations and the fact that a purposeful sample was selected, data from the present study are not readily generalisable. However, care was taken to select schools and students that mirror the population groups in the area, and therefore the data allow valuable insights into the needs of students regarding school-based HCT.

One of the most important contributions that the present study could make is to afford youth a voice which has been lacking in discussions about the provision of HCT in schools. It should further contribute to the debate around the ethics and feasibility of providing HCT in South African schools, and can be used by governmental and NGO providers of HCT in schools to provide a youth-friendly service. Lastly, the present research adds to the limited existing body of literature regarding models of HCT provision to youth.

If HCT is to be provided in schools, service providers need to (1) address students’ concerns regarding privacy and confidentiality, (2) provide information regarding HCT to students before HCT takes place, (3) ensure that staff are experienced and trained to work with youth and (4) that students who test positive are followed up and supported.
